# Shotgun Bisulfite Sequencing of the *Betula platyphylla* Genome Reveals the Tree’s DNA Methylation Patterning

**DOI:** 10.3390/ijms151222874

**Published:** 2014-12-10

**Authors:** Chang Su, Chao Wang, Lin He, Chuanping Yang, Yucheng Wang

**Affiliations:** 1 State Key Laboratory of Tree Genetics and Breeding, Northeast Forestry University, No. 26 Hexing Road, Xiangfang District, Harbin 150040, China; E-Mails: changsu1989@gmail.com (C.S.); wangchao7993@126.com (C.W.); helin8565@126.com (L.H.); yangcp@nefu.edu.cn (C.Y.); 2 Xinjiang Institute of Ecology and Geography, Chinese Academy of Sciences, 818 South Beijing Road, Urumqi 830011, China

**Keywords:** *Betula platyphylla*, plant, epigenetic, DNA methylation, BS-Seq

## Abstract

DNA methylation plays a critical role in the regulation of gene expression. Most studies of DNA methylation have been performed in herbaceous plants, and little is known about the methylation patterns in tree genomes. In the present study, we generated a map of methylated cytosines at single base pair resolution for *Betula platyphylla* (white birch) by bisulfite sequencing combined with transcriptomics to analyze DNA methylation and its effects on gene expression. We obtained a detailed view of the function of DNA methylation sequence composition and distribution in the genome of *B. platyphylla*. There are 34,460 genes in the whole genome of birch, and 31,297 genes are methylated. Conservatively, we estimated that 14.29% of genomic cytosines are methylcytosines in birch. Among the methylation sites, the CHH context accounts for 48.86%, and is the largest proportion. Combined transcriptome and methylation analysis showed that the genes with moderate methylation levels had higher expression levels than genes with high and low methylation. In addition, methylated genes are highly enriched for the GO subcategories of binding activities, catalytic activities, cellular processes, response to stimulus and cell death, suggesting that methylation mediates these pathways in birch trees.

## 1. Introduction

Cytosine DNA methylation is an epigenetic mark that is important in silencing transposons and other repetitive sequences, and has effects on diverse biological processes, including genomic imprinting, X-chromosome inactivation, and the expression of endogenous genes [1,2,3,4]. According to the sequence context of the cytosines, DNA cytosines can be classified into three types,* i.e.*, CG, CHG, and CHH (H = A, G or T). In plants, methyltransferase 1 (MET1) maintain CG methylation by acting on hemi-methylated DNA after replication at symmetric sequence contexts. CG methylation is found in both genes and repeats, and is involved in gene expression regulation [5]. CHH methylations, and some CHG methylations, are generally maintained by domains rearranged methyltransferase (DRM1/DRM2), which are involved in *de novo* methylation of DNA [6]. The plant-specific protein chromomethylase 3 (CMT3) maintain high levels of CHG methylation [7]. The two non-CG methylation types, CHG, and CHH, are mostly absent from genes and are mainly found in intergenic, repeat-rich regions of the genome and play a critical role in silencing transposons [8].

Sodium bisulfite converts unmethylated cytosines to uracils, but 5-methylcytosines remain unconverted. Hence, after polymerase chain reaction (PCR) amplification, unmethylated cytosines appear as thymines and methylated cytosines appear as cytosines [9]. Extensive research in herbaceous plants has described different DNA methylation patterns in different species and their functions and distributions [10,11,12]; however, there has been little work done on the DNA methylation of forest species, especially trees. Recently, Feng *et al.*, compared DNA methylation among eight plant and animal species using bisulfite sequencing technology, and briefly reported the genome methylation patterns for *Populus trichocarpa* [13]. However, in that study, the *Populus* methylome had relatively low sequencing coverage, and was not sufficient to quantify the level of methylation of individual cytosines [13].

Forests cover 30% of earth terrestrial surface; they protect of biodiversity, and are the producers of the biosphere [14]. Forest trees are different to herbaceous plants in various ways. For instance, forest tree populations have evolved under more selective pressures than annual herbaceous plants. Trees show more extensive secondary growth, including the obvious growth and development of secondary xylem, compared with herbaceous plants. Although some plant methylation maps have been acquired, a high resolution, accurate profile of forest tree species is urgently to allow the exploration of the characteristics of woody plants.

Here, we developed a single base-resolution map of a forest species, *Betula platyphylla* (white birch), using bisulfite-based detection of methylated cytosines with high-throughput sequencing (Bisulphite Sequencing or BS-seq). White birch is widely distributed in Eurasia, can grow on infertile soil and is resistant to low temperature. With its rapid growth, white birch is a pioneer species of afforestation. In this study, we generated DNA methylation and transcriptomic profiles for the current year secondary xylem tissues. Our goal was to describe the cytosine DNA methylation pattern in birch and provided useful data for future studies on birch epigenetics.

## 2. Results and Discussion

### 2.1. Generation of Methylation Data of B. platyphylla

To generate a DNA methylation map across the genome of birch with the current year secondary vascular tissues, shotgun sequencing of bisulfite-treated birch genomic DNA was performed using Illumina sequencing technology (Illumina GA). After bisulfite conversion of the birch genome, genomic DNA libraries were constructed and sequenced. 178.02 million raw reads were generated (Table 1). To ensure the accuracy of the sequencing, filters were used to retain the reads mapping to sequences that are unique in the genome after bisulfite conversion from every possible methylation pattern. This resulted in a conservative dataset of 148.78 million effective reads. Thus, the sequence yield for final analysis was 13.08 gigabase pairs (Gb), which covered 83.58% of the reference *B. platyphylla* genome, with an average depth of 30-fold for the whole genome (Table 1). We used the unmethylated chloroplast genome [15] to calculate the sum of the non-conversion rate and T-C sequencing error rate, which was low (0.42) for the sample, indicating a high conversion rate and reliable data.

**Table 1:** Summary of sequencing results and reads alignment.

Raw Reads (M)	Raw Base Number (Gb)	Effective Reads (M)	Effective Base Number (Gb)	Genome Coverage (%)	Average Depth Per Base and Strand (X)
178.02	15.65	148.78	13.08	83.58	15.02

### 2.2. Analysis of Percentages of Methylated Cytosines (mCs) in CG, CHG and CHH

**Figure 1:**
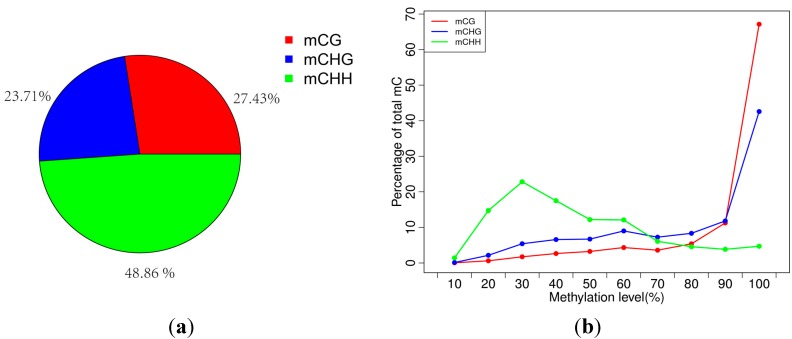
DNA methylation pattern in *Betula platyphylla*. (**a**) Relative percentages of methylcytosines (mCs) in the sequence contexts of CG, CHG (where H = A, C or T), and asymmetric CHH; (**b**) Distribution of methylation level of mCs in each sequence context. Only mCs covered by at least five reads were used to calculate the methylation level. The *x*-axis was defined as the percentage of reads showing mCs at a reference cytosine site. The *y*-axis indicates the fraction of total mCs calculated within bins of 10%.

Methylation in *B. platyphylla* exists in three sequence contexts, *i.e.*, CG, CHG (where H = A, C or T), and asymmetric CHH [1]. The percentages of methylated cytosines (mCs) in CG, CHG and CHH contexts were 27.43%, 23.71% and 48.86%, respectively (Figure 1a). The average methylation level in CG, CHG and CHH were 42.64%, 28.80% and 5.16%, respectively, with the methylation level being defined as the proportion of reads showing mC among all reads covering the same cytosine site. In birch, there was tendency toward highly methylated CG sites methylated (most CGs were either unmethylated or highly methylated (80%–100%)), CHG showed a more uniform distribution between (20%–100%), and CHH sites showed a low level of methylation (were either unmethylated or methylated at 20%–30%) (Figure 1b). These trends were similar to those of *Populus trichocarpa* (percentages of CG, CHG and CHH are 41.9%, 20.9% and 3.25%, respectively) [13] rather than those of *A. thaliana* (percentages of CG, CHG and CHH are 24.60%, 6.98% and 1.70%, respectively) [5,6,16]. The methylation level of CG and CHG are similar in these two tree species, but the methylation level of CHH in birch (5.16%) was almost twice that in popular. This result suggested that the DRM2 methyltransferase, which maintains non-CG methylation in plants [7,17], may have higher expression level in birch than in popular.

### 2.3. Analysis of the Methylation Profiles

**Figure 2:**
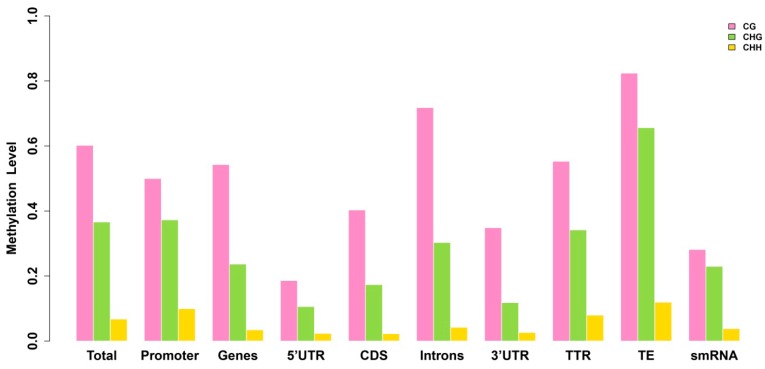
Relative methylation level in each sequence context for different genomic regions. The sequence contexts are shown in the *x*-axis, and the *y*-axis shows the methylation level.

We further analyzed the methylation profiles of the promoters (2 kb upstream), genes, 5'-Untranslated Regions (5'-UTR), Coding Sequences (CDS), Introns, 3'-Untranslated Regions (3'-UTR), Transcriptional Termination Region (TTR) (2 kb downstream), transposable elements (TEs) and small RNAs (including miRNA, rRNA, snRNA, tRNA) (Figure 2, Table 2). For accuracy, at each reference cytosine, the methylation levels (mC/C ratio) of at least five reads were required (Table 3). There was an obvious enrichment of methylation in TEs, and 5'-UTR had the lowest methylation level. The enrichment of methylation in TEs reflects the fact that DNA methylation is an important epigenetic event used by higher eukaryotes to regulate silencing of repetitive elements, and high methylation over TE sequences serves as a self-defense mechanism in case of transcription of TEs [18]. The lower methylation of 5'-UTRs may provide a high probability for genomic loci to be transcribed.

**Table 2:** DNA methylation patterns in different genomic regions.

Methylation Pattern	Total	Promoter	TTR	Genes	CDS	Introns	5'-UTR	3'-UTR	TE	smRNA
ML	0.1728	0.1864	0.1674	0.1248	0.1051	0.1428	0.0607	0.0712	0.2759	0.1164
MD	0.4276	0.3829	0.4122	0.4800	0.5454	0.4465	0.4264	0.4777	0.4071	0.4218

ML: relative methylation level; MD: methylation density; TTR: Transcriptional Termination Region (2 kb downstream); CDS: Coding Sequences; UTR: Untranslated Regions; TEs: transposable elements.

**Table 3:** DNA methylation pattern in *Betula platyphylla* at difference depths.

Pattern	mCG	mCHG	mCHH
Methylation level (depth ≥ 1×)	42.64	28.8	5.16
Methylation level (depth ≥ 5×)	60.19	36.63	6.79

**Figure 3:**
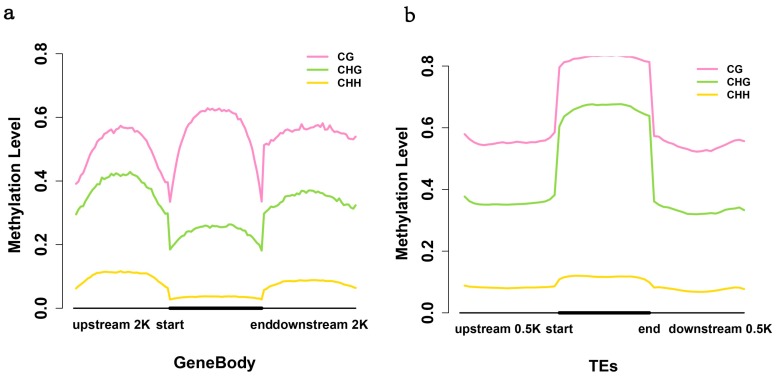
Methylation level of transposable elements (TEs), gene body and its upstream and downstream sequences. (**a**) Methylation levels of the gene body and its upstream and downstream sequences. Two-kilobase regions upstream and downstream of each gene were divided into 50 bp intervals and analyzed. Each gene body was divided into 40 intervals for analysis; (**b**) Methylation level of TEs. TEs and 0.5 kb flanking sequences regions on both sides were analyzed. Each TE was divided into 20 intervals.

We further calculated methylation levels in the context of gene bodies and 2 kb of their upstream and downstream regions (Figure 3a). Boundaries between gene bodies and flanking DNA showed a sharp drop in methylation; however, DNA methylation extended from TEs into the flanking DNA, showing a more gradual reduction (Figure 3b). This trend of methylation level was similar to that of *Arabidopsis thaliana*, *Populus trichocarpa*, *Japonica* and *Oryza sativa*, and may be a common characteristic of flowering plants (*Chlamydomonas reinhardtii* shows a different pattern) [13,19]. The methylation level of birch exhibited a characteristic peak in the gene bodies, which was also observed genome-wide in *A. thaliana*, *P. trichocarpa*, *Japonica* and *Oryza* [13,20]. However, there appears to be some “CHG-gene body” methylation, which seems to be unique to birch. Although the function of this gene body methylation remains unknown, it has been hypothesized that it suppresses spurious transcription from cryptic promoters that might otherwise interfere with gene regulation [13,21].

**Figure 4:**
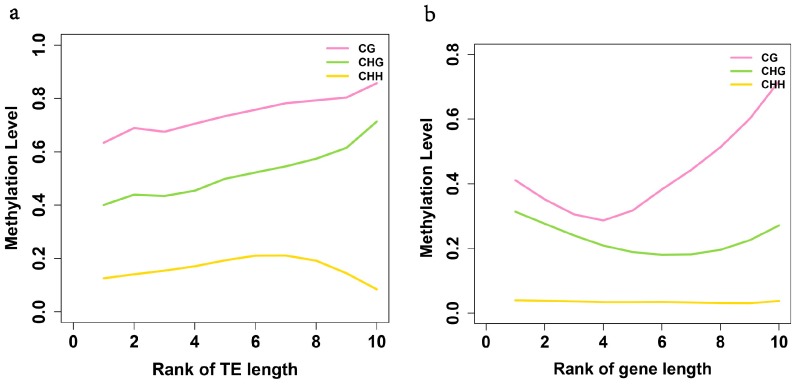
Analysis of the relationships between methylation level and sequence length. The relationships between methylation level and sequence length in TE regions (**a**) and genes (**b**).

In addition, we found a positive correlation between sequence length and methylation density for TEs (Figure 4a), but not for genes (Figure 4b), which is different to the case in *A. thaliana*, where sequence length and methylation density are positively correlated for both genes and TEs [5]. This may be related to the fact that a large number of genes in the birch genome are relatively shorter than those in Arabidopsis and these genes are almost all highly methylated in white birch compared with Arabidopsis (Figure S1).

### 2.4. Exons Have Lower Methylation Levels than Introns in Birch Genes

**Figure 5:**
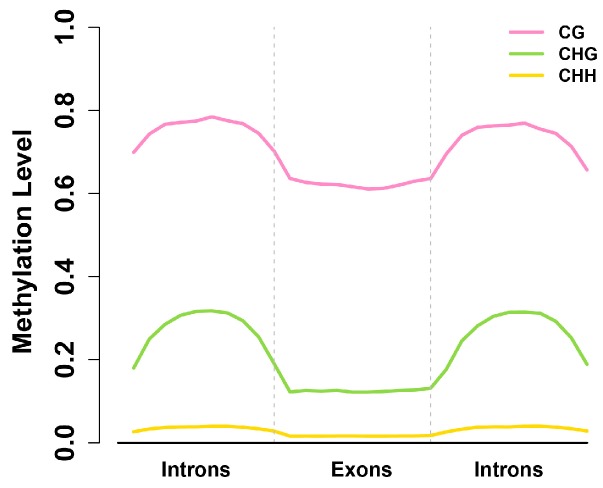
Methylation levels across exons and introns. Only internal exons (flanked by introns on both ends) that did not contain any 5'- or 3'-UTR (Untranslated Regions) bases were used. Upstream and downstream regions of exons for analysis were a similar length to the exon. Two vertical blue lines mark the intron–exon and exon–intron boundaries.

To examine gene body methylation, we further explored methylation levels across introns and exons (Figure 5; Table 2). Strikingly, we found that methylation level in introns was higher than in exons, which is opposite to *A. thaliana*, *P. trichocarp*, *and O. sativa* [13]. This high enrichment of methylation in introns may have a direct or indirect role in the regulation of transcription in birch. To analyze the whole methylation trend, all the coding genes of birch were classified into seven transcription elements, including upstream, first exon, first intron, internal exon, internal intron, last exon, and downstream. The result showed that methylation level of CG, CHG and CHH contexts in exons were all lower than in those in introns (Figure S2). This trend was opposite to that of *Arabidopsis*, Rice, poplar, and *Ciona*, which showed clear enrichment of methylation in exons [13].

### 2.5. There Is no Correlation between Sequence Context and Methylation Preference in Birch

**Figure 6:**
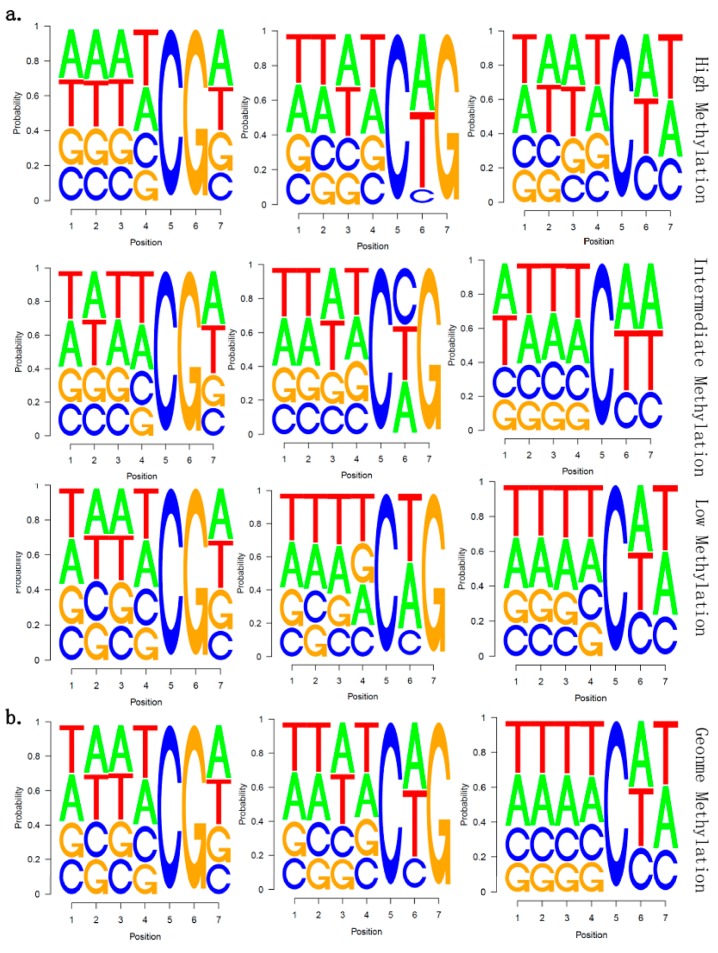
Sequence preferences for methylation in CG, CHG, and CHH contexts. (**a**) The mC site methylation level was divided into three groups: High Methylation (ML ≥ 0.8), Intermediate Methylation (0.2 < ML < 0.8) and Low Methylation (ML ≤ 0.2) for 7-mer sequences in which the methylated cytosine is in the fifth position; (**b**) all genomic7-mers in whole genome were analyzed. ML indicated methylation level.

In some plants, methylation preference is found to correlate with sequence context [5,21]. To reveal whether there was a relationship between sequence context and methylation preference in birch, we calculated the methylation percentage of all possible 7-mer sequences in which the methylated cytosine was in the fifth position (allowing an analysis of four nucleotides upstream of CG, CHG, and CHH methylation). The result showed that there was no obvious sequence context specificity in white birch, indicating that there is no correlation between sequence context and methylation preference (Figure 6). This trend is not consistent with a previous observation in *A. thaliana* [5], in which the lowest CG methylation sequences were highly enriched for the sequence ACGT; poorly methylated CHG sites were depleted of upstream cytosines, but tended to contain cytosine following the methylated base (which was similar to the trend in wheat germ DNA sequences [22]); and highly methylated CHH sequences showed a tendency for cytosines, CG dinucleotides were present upstream and the sequence TA followed the methylated cytosine. In contrast, poorly methylated CHH sequences contained a cytosine following the methylated cytosine, and frequently contained a cytosine, but always lacked an adenine, two nucleotides downstream [5].

### 2.6. Methylation and Gene Expression

To reveal the functional consequences of gene body methylation, we generated the transcriptome of genome-wide genes for two biological replicates of samples (sample A and B), using Illumina sequencing techniques. In total, we obtained 25,647,400 and 26,811,962 clean reads from the two samples. After filtration, Q20 percentages of the two samples both reached 98% (Table S1), and there was a good correlation between the sample A and B (*R*^2^ = 0.976) (Figure S3), indicating that consistent results were produced from these two samples.

**Figure 7:**
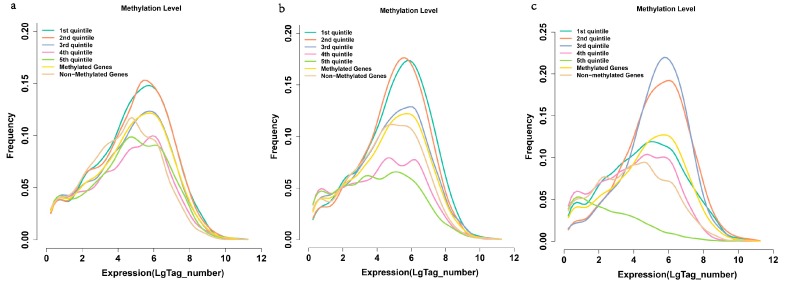
Correlation between DNA methylation and expression levels of genes. (**a**) Expressions of genes with methylated promoters compared with genes with unmethylated promoters; (**b**) Expression of genes with methylated transcription termination regions (TTRs) compared with genes with unmethylated TTRs; (**c**) Expression of methylated compared with unmethylated genes. Genes were rank-ordered based on promoter, TTS, gene body methylation level and divided into quintiles. The first quintile is the lowest and the fifth is the highest.

We then used the data of transcriptome analysis and methylation level (in promoter, gene body and TTR) to analyze the relationship between methylation and gene expression. Genes were categorized into unmethylated and methylated groups, and the methylated ones were further divided into five groups according to their relative methylation levels (with 20% intervals). We chose the genes at 3 to 8 (in *x*-axis) for further study, because the genes in other parts have low frequencies (Figure 7a,b). The results showed that the repression effect is weak for the slightly and moderately promoter- and TTR-methylated genes; while the repression effect is strong for the heavily methylated ones from 3 to 8 in the *x*-axis (Figure 7), in which, slightly methylated ones have the largest fraction of genes to express. This confirmed the well-known gene regulatory mechanism that promoter and TTRs methylation play an essential role in gene expression. We observed that there is no obvious difference in frequencies among the different gene methylation levels at the first expression level (0–1 in *x*-axis). After a certain point, heavy gene-body methylation appears to repress gene expression (Figure 7c), and the genes with moderate levels of body methylation tend to have the higher frequency to express than the slightly and heavy gene body methylated genes (Figure 7c). These observations are consistent with previous studies in rice [19,23] and *A. thaliana* [6,16]. It has been showed that gene-body methylation play a role in preventing transcriptional initiation from cryptic sites within genes, but at the cost of impending transcriptional elongation [16], which might lead to the observation that moderately body-methylated genes have the highest frequency of expression.

**Figure 8:**
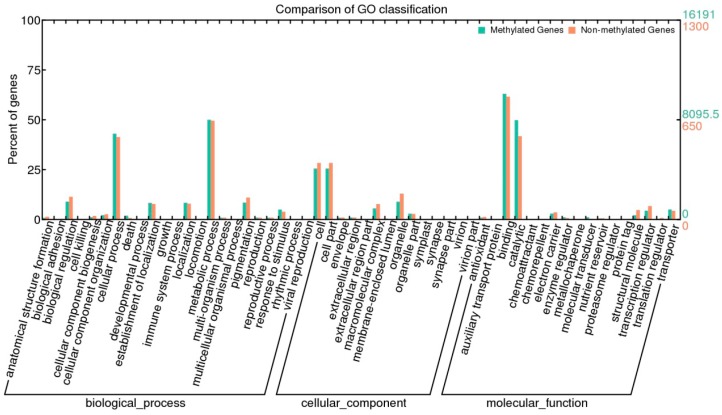
Analysis of methylated and unmethylated genes using WEGO (Web Gene Ontology Annotation Plotting) analysis. Annotations are grouped into the terms of cellular components, molecular function or biological process based on the *Betula* GO annotation information (unpublished). Gene numbers and percentages (on the log scale) are listed for each category.

We further used the BGI WEGO (Web Gene Ontology Annotation Plotting) [24] to categorize the methylated and unmethylated genes functionally. GO analysis indicated that methylated genes tended to be enriched in the GO terms of binding activities and catalytic activities (Figure 8). For biological processes, methylated genes were mainly associated with cellular processes, response to stimuli and cell death (Figure 8). In contrast, unmethylated genes were mainly enriched in transcription regulators and functions associated with biological regulation and pigmentation processes. These results suggested that DNA methylation plays a direct or indirect role in binding and catalytic activities and the pathways of in response to stimulus and cell death; however, it has a relatively lower effect on transcription, biological regulation and pigmentation processes [25].

## 3. Experimental Section

### 3.1. Plant Materials

Secondary xylem tissues were collected from a four-year-old wild-type birch in Harbin, China. DNA was isolated from secondary xylem tissues by the CTAB method, and RNA was isolated using a modified CTAB (cetyl trimethylammonium bromide) method [26].

BS-Seq (Bisulphite Sequencing) libraries construction and sequencing. Genomic DNA was fragmented by sonication, using a Diagenome sonicator, to a mean size of approximately 200–300 bp, followed by blunting, addition of dA (Adenine deoxyribonucleotides) to the 3'-end, and adaptor ligation according, to the manufacturer’s instructions (Illumina, San Diego, CA, USA). Bisulfite conversion of birch DNA was carried out using a ZYMO EZ DNA Methylation-Gold kit with EpiMark HotStart Taq (NEB, Ipswich, MA, USA). The resultant DNAs were subjected to paired-end sequencing with the read length of 44 or 75 nt for each end, using the ultrahigh throughput Illumina Hiseq 2000 according to the manufacturer’s instructions [19,27].

### 3.2. Mapping and Processing of Bisulphite Sequencing (BS-Seq) Reads

All reads from the sample were mapped to the birch genome sequence, which is available online [28]. DNA methylation has strand specificity; therefore, the plus strand and the minus strand of *B. Platyphylla* genome should be separated and used as different alignment target sequences for BS-Seq reads. Thus, each cytosine in reference genome sequences was converted to thymine, termed the T-genome, which represents the plus strand. Meanwhile, each guanine in reference genome sequences was converted to adenosine, termed the A-genome, which represents the minus strand. To map the raw 44 or 75 nt pair-ended BS-Seq reads, the original reads were computationally converted to the alignment forms by the following steps: (1) observed cytosines on the forward read of each read pair were in silico replaced by thymines; (2) observed guanines on the reverse read of each read pair were in silico replaced by adenosines.

We used the software SOAPaligner [29], allowing up to two mismatches for 44 nt reads and four mismatches for 75 nt reads to map the computationally transformed reads to the alignment target sequences. Multiple reads mapping to the same start position were regarded as clonal duplication, which might be generated during PCR process, and only one of them was retained. For mC detection, we transformed each aligned read and the two strands of the birch DNA back to their original forms to build an alignment between the original forms. Cytosines in the MethylC-seq reads that also matched the corresponding cytosines in the plus (Watson) strand, or guanines in the MethylC-seq reads that matched the corresponding guanines in the minus (Crick) strand were regarded as potential mCs. The Q score, which is used in the base-calling pipeline (Illumina) to detect sequences from the raw fluorescent images, was calculated as:

*Q* = 10log10 [*p*(*X*)/(1 − *p*(*X*)] (1)

where *p*(*X*) is the probability that a read is correctly called. We then carried out a filtering process to filter out all potential mCs with *Q* scores less than 20, guaranteeing that a base would be correctly called at more than 99% probability, which is highly conservative for calling reliable bases [19,24].

### 3.3. Construction of a cDNA Library and Illumina Sequencing

Total RNA was isolated from birch using the CTAB method, and mRNA was isolated by total RNA Oligo (dT) magnetic beads adsorption, and sheared into small fragments for synthesis of cDNA. cDNA was synthesized using mRNA fragments as templates and random primers. To create blunt ends, the synthesized cDNAs were digested with T4 DNA polymerase and Klenow DNA polymerase such that the 3' to 5' exonuclease activity of these enzymes removed the 3' overhangs and the polymerase activity filled in the 5' overhangs. The polymerase activity of the Klenow fragment added an adenine “A” base to the 3' ends of the blunt phosphorylated DNA fragments. The DNA fragments with “A” overhang ends were ligated with specific adaptors equipped with a single thymine “T” base overhang at their 3' end. The fragments were electrophoresed through an agarose gel and fragments of about 200 nucleotides were identified, gel purified, and PCR amplified using a primer set the specifically anneals to the ends of the “T” adapters. The PCR products were purified using the QIAquick PCR Purification Kit (QIAGEN, Hilden, North Rhine-Westphali, Germany) and the size, purity, and concentration of the constructed library were evaluated.

Transcriptome sequencing was conducted using Illumina RNA-seq. An Illumina HiSeqTM 2000 (Illumina, San Diego, CA, USA) generated the raw PE reads. After filtering of low quality reads, the remaining high-quality RNA reads were aligned to the birch genome using Bowtie, with perfect matches. Reads aligned to multiple locations in the birch genome were not included in the analysis.

## 4. Conclusions

In conclusion, we generated a high depth single base-resolution methylome of the birch tree. The percentages of mCs in CG, CHG and CHH contexts were 27.43%, 23.71% and 48.86%, respectively. The methylation level of birch exhibited a characteristic peak in the body of protein-coding genes. There was a positive correlation between sequence length and methylation density for TEs, but not for genes. In addition, some distinct methylation phenomena that are different to those observed previously in other plants are found; *i.e.*, in birch, the methylation level in introns was higher than in exons, and there was no relationship between sequence context and methylation preference. Our study also showed that DNA methylation in birch is involved in binding and catalytic activities, cellular processes, responses to stimuli and cell death.

## Supplementary Materials

Supplementary materials can be found at http://www.mdpi.com/1422-0067/15/12/22874/s1.
